# Modification of Pyrolytic Oil from Waste Tyres as a Promising Method for Light Fuel Production

**DOI:** 10.3390/ma12060880

**Published:** 2019-03-15

**Authors:** Cezary Dębek

**Affiliations:** Department of Elastomers and Rubber Technology, Institute for Engineering of Polymer Materials and Dyes, Harcerska 30 Street, 05-820 Piastów, Poland; c.debek@ipgum.pl

**Keywords:** tyre pyrolysis, rubber, pyrolytic oil, hydrorefining, hydrodesulphurization, light fuel oil

## Abstract

Due to its high total sulphur content and other unfavourable properties, pyrolytic oil obtained as a result of tyres pyrolysis is not suitable for use as motor or heating fuel. Therefore, pyrolytic oil was hydrorefined. Hydrorefined oil was used as a component of light heating oil. A composition was prepared from 30 wt % hydrorefinate with 70 wt % Ekoterm Plus (a commercial oil). Unfortunately, the flash point temperature of the hydrorefinate was too low, and did not allow fuel compliant with the Polish standard PN-C-96024:2011 for L1 light heating oil to be obtained. Therefore, the fraction with boiling point below 180 °C was removed from the hydrorefinate. The residue, with a flash point of 74 °C and a sulphur content of 0.143 wt %, was mixed with Ekoterm Plus and fuels with a hydrorefinate fraction content of 30 and 50 wt % were prepared. The composition containing 30 wt % met the requirements for L1 oil in the whole range of tested parameters. Total sulphur content was 0.092 wt %, specific weight was 856 kg/m^3^ and closed cup flash point was 64 °C. However, the composition containing 50 wt % hydrorefinate did not meet the requirements regarding sulphur content and specific weight. Sulphur content, specific gravity, and flash point are the parameters limiting the possibility of using hydrorefined pyrolytic oil for composing light heating oils compliant with the mentioned standard.

## 1. Introduction

The tyre is a composite rubber product which accounts for the bulk of rubber production. The rubber used in tyres consists of approximately 60–65 wt % of various raw rubbers, 25–35 wt % of technical carbon black and 0–5 wt % of silica, plasticizers and anti-aging substances. Rubbers are cross-linked by means of sulphur cure systems containing sulphur, an accelerator, usually an organic-sulphur compound and an activator, which usually consists of zinc oxide and stearic acid. In addition, tyres contain steel reinforcements (cord and wire) and textile reinforcements (usually polyamide cords). The production of tyres accounts for about 60%–70% of the rubber industry production, and the percentage of tyres in the collection of used rubber products is even higher, amounting to about 80 wt % [1,2,3,4,5,6].

Like other rubber products, used tyres do not decompose easily, which is why their disposal poses many problems and requires a great deal of energy and time. Therefore, the basic and cost-effective way to dispose of rubber scrap—especially used tyres—is still burning, usually in cement plants [4,5]. However, for environmental reasons, incineration is limited by the Waste Incineration Directive [4,6].

One of the methods of disposing of used tyres or other rubber waste is their pyrolysis. This is a process of the thermal decomposition of organic substances (i.e., in the case of rubber, macromolecules of cross-linked rubber), without oxygen or oxidizing substances [1,2,3,4,5].

The pyrolysis of tyres produces four products: gas and pyrolytic oil formed by cracking macromolecules and other organic substances, steel from cord and bead wire, and pyrolytic coal, which is mainly recovered carbon black, used in the production process, modified and contaminated with mineral substances and sludge [1,2,3,4,5,7,8,9,10,11,12].

For economic reasons, pyrolytic oil is usually considered as the most important or at least the most easily sellable product on the market [4,5]. Depending on the type of tyres used for pyrolysis and the parameters of the oil process, it is usually obtained in quantities of about 35–50 wt % [1,2,3,4,5,7,8,9,10,11,12].

Pyrolytic oil obtained, for example, as a result of the periodical low-temperature (350–450 °C) pyrolysis of whole car and van tyres is a dark, unclear, viscous liquid with a rather intense smell. It consists of saturated and unsaturated linear and cyclic hydrocarbons, as well as aromatic hydrocarbons, resinous substances and solid particle drifts. Since tyres are made of rubber cross-linked with sulphur vulcanization systems, pyrolytic oil contains significant amounts of sulphur (i.e., over 1 wt %), which disqualifies its use as, for example, engine or heating fuel—both in terms of light and heavy oils [4,10,13].

Despite its high sulphur content, limited stability and other unfavourable features, in Poland pyrolytic oil is sometimes used to compose heating fuels due to its significantly lower price. For example, the mixing of pyrolytic oil with crude rapeseed oil at the weight ratio of 1:1 reduces the sulphur content to the level meeting the requirements for heavy fuel oils according to the Polish standard [13], which allows sulphur content up to 1 wt %. Additionally, mixing with rapeseed oil weakens the aromatic character of pyrolytic oil. The advantageous feature is the lack of vanadium, which eliminates the corrosive effect of oil on heating installations. Thus, obtaining standardized heating fuel is simple but is limited to heavy oil, the combustion of which is possible only in appropriate heating installations [10].

In addition to energy applications, the possibilities of using heavier oil fractions as plasticizers in rubber materials are also being investigated. This is an interesting use of pyrolytic oil as a raw material [7,8].

In recent years the technology of the pyrolysis of tyres and other used rubber products has been implemented in Poland in approximately a dozen plants. These are small companies, processing 10–40 Mg of rubber waste per day. There are also known companies processing polyolefin wastes or other plastics. Most of them produce crude pyrolytic oil, without refining processes. This oil is mainly sold to companies dealing with the blending of heating fuels. After mixing with heavy oils, for example, it is burnt in appropriate installations [4,5,14].

The main problem limiting the use of pyrolytic oil as an energy source is the high content of total sulphur, even above 1 wt %, which makes it impossible to use this oil for the composition of light heating oils [1,2,3,4,9,10,12,15], for which (according to the Polish standard) a sulphur content of up to 0.1 wt % is allowed [13].

Apart from high total sulphur content and low flash point, another problem is a significant content of unsaturated hydrocarbons limiting the stability of pyrolytic oil. Therefore, many methods of sulphur removal and saturation of unsaturated compounds, which are reviewed in [2,7,12], are being investigated.

The most effective method is the hydrorefination of pyrolytic oil. In Reference [4], using relatively drastic conditions of this process (hydrogen pressure 3 MPa, 380 °C, NiMo–Al_2_O_3_ catalyst), it was possible to obtain hydrorefined pyrolytic oil with total sulphur content even below 0.1 wt %. This value meets the requirements of the Polish standard [13] for light fuel oil L1 type. Hydrorefination is essentially the only method used industrially, and allows the sulphur content to be lowered below 0.1%. In addition, there is a reduction in unsaturated compounds that contain other heteroatoms.

Han et al. [15] describe the results of hydrotreating the mixture of vegetable oil and tyre pyrolysis oil with CoMo/Al_2_O_3_ catalyst. As a result of hydrotreating, the total sulphur content was reduced to about 0.2 wt %.

Thanks to total sulphur contents even below 0.1 wt %, hydrotreated oil obtained as a result of the pyrolysis of tyres and other used rubber products may be used for composing light heating oils meeting the above-mentioned standard. The possibility of composing light heating oils opens up a much larger market than in the case of heavy oil. Therefore, this paper presents the results of research on the basic properties of hydrorefined crude pyrolytic oil obtained as a result of the pyrolysis of car tyres and heating fuels produced by mixing Ekoterm Plus fuel oil with hydrorefined oil.

## 2. Materials and Methods

Hydrorefined pyrolytic oil (HPO) was obtained by hydrorefining crude pyrolytic oil (CPO) obtained as a result of pyrolysis of unsorted whole car tires, size R14-R18, more precisely described in [4,10]. The hydrogenation process was carried out in the presence of a nickel–molybdenum catalyst (Akzo Nobel) deposited on alumina carrier (NiMo–Al_2_O_3_) at the temperature of 380 °C and hydrogen pressure of 3 MPa. The raw material was distilled before hydrorefining in order to prevent inactivation of the catalyst by impurities present in the CPO. The catalyst properties, apparatus used and the exact method of hydrogenation are described in [4].

The fraction of hydrorefinate (FHPO) obtained by distilling the gasoline fraction had a boiling point below 180 °C.

We used light heating oil of L1 class, trade name Ekoterm Plus (EP), produced by Orlen S.A. (Płock, Poland). The properties of HPO, FHPO, EP and, for comparison, CPO are shown later in this paper. The following research methods were used.

(a) Density was determined according to the method defined in EN ISO 3675:2004.
(b) Kinematic viscosity was determined according to the method defined in the PN-EN ISO 3104:2006 standard.
(c) Fractional composition was determined by distillation under atmospheric pressure according to the PN-EN ISO 3405:2006 standard, using the HDA 620 (Walter Herzog GmbH, Lauda-Königshofen, Germany), at a distillation rate of 5 mL/min.
(d) Cetane index (CI) was calculated according to PN-EN ISO 4264:1996.
(e) Flash point was determined in Pensky–Martens closed cup test, according to PN-EN ISO 2719:2007 and in an open cup using the Cleveland method according to PN-EN ISO 2592: 2017–10.
(f) Pour point was determined according to ASTM D97.
(g) Sulphur content was determined according to PN-EN ISO 20847: 2007/Ap1: 2014–02 by X-ray fluorescence using the Thermo Electron ARL Advant’XP Uniquantometer wave-dispersive spectrometer (Thermo Electron Corporation, Waltham, MA, USA).
(h) Analysis of the hydrocarbon composition of the oil was performed by means of gas chromatography coupled with mass spectrometry (HP6890/TCD/FID, HP6890/MSD, column HP5MS 30 m × 0.25 µm × 0.25 µm, Hewlett-Packard, Palo Alto, CA, USA).
(i) Heat of combustion was designated PN-86/C-04062 using an automatic calorimeter KL-10 made by Precyzja (PPHU Precyzja, Bydgoszcz, Poland).

## 3. Results and Discussion

**Figure 1:**
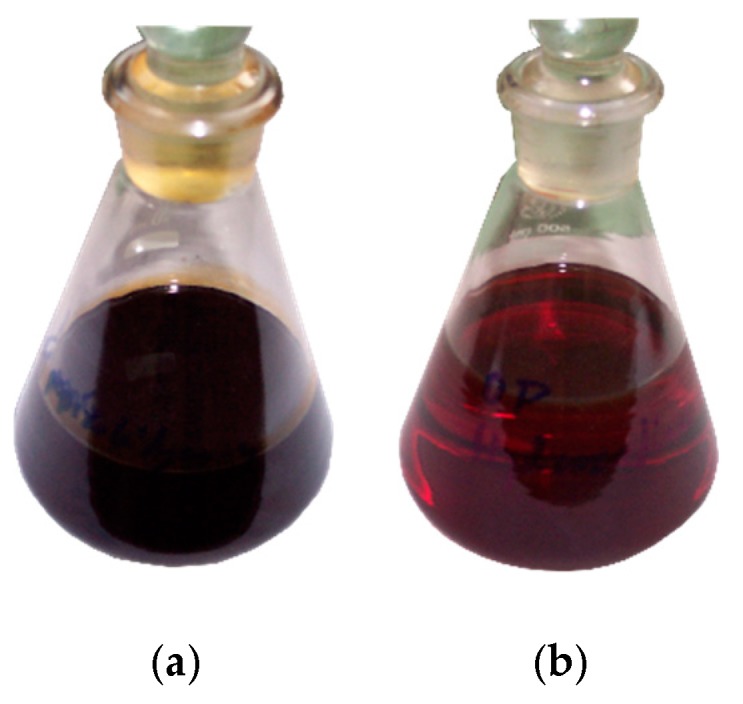
Pictures of (**a**) crude pyrolysis oil (CPO) and (**b**) hydrorefined oil (HPO).

Due to its high total sulphur content and other unfavourable properties, CPO obtained as a result of tyre pyrolysis is not suitable for use as motor or heating fuel. Therefore, pyrolytic oil with total sulphur content of 1.102 wt % was hydrorefined in the presence of a nickel–molybdenum catalyst. HPO with sulphur content of 0.145 wt %, double-reduced content of unsaturated hydrocarbons and significantly reduced content of aromatic compounds was obtained. Pictures showing the appearance of CPO and HPO are shown in Figure 1.

Visually, CPO is a dark, dense liquid with an intense smell. It contains a significant amount of solid impurities, carbon black and mineral particles and resinous substances forming a gel sludge [4,10]. After the hydrorefination process, with prior distillation to remove impurities, the oil became transparent and changed colour to light brown. The hydrocarbon compositions of HPO and CPO are shown in Table 1.

CPO contains as much as 48 wt % of aromatic hydrocarbons, which is a serious shortcoming of CPO, limiting the scope of application as a fuel to industrial equipment only. The content of saturated alkanes and cycloalkanes was in total about 17 wt %. There was a high content of unsaturated compounds—both linear and cyclic—of about 11 wt %.

The hydrorefining of CPO reduced the content of aromatic compounds from 47 to 35 wt %, which is similar to the level observed in light fuel oils. The content of saturated hydrocarbons (linear and cyclic) increased significantly from about 17 to 37 wt % The content of unsaturated hydrocarbons was reduced by half, from 11 wt % in the case of CPO to 5.5% for HPO, improving the storage stability of HPO.

The basic physicochemical properties of HPO and for comparison of CPO and commercial light heating oil (EP as well as FHPO (i.e., HPO devoid of gasoline fraction)) are presented in Table 2.

Due to the unfavorable properties including too high density, low flash point in a closed cup, fractional composition and very high sulphur content CPO differs significantly from the requirements for fuel oils. The high sulphur content is a consequence of the vulcanization of rubber compounds used in the production of tyres. Therefore, CPO cannot be used for the composition of light fuel oils, but it can be used to obtain heavy fuel oil compliant with the Polish standard, for which the upper sulphur content was determined at the level of 1 wt % [13].

Hydrorefining of CPO against NiMo–Al_2_O_3_ catalyst at 380 °C and hydrogen pressure of 3 MPa allowed the sulphur content to be reduced from 1.102 wt % to 0.145 wt %. The sulphur content in HPO was higher than reported in Reference [4], but the raw material then used also contained less total sulphur.

Of course, due to its sulphur content above 0.1 wt %, high density of 874 kg/m^3^ and low cetane index (37.4), HPO cannot be used for composing diesel fuels. It can potentially be added in appropriate proportions as a component of light heating oil.

Commercial light fuel oil (EP) contained 0.072 wt % sulphur. The combustion heat of 40.3 MJ/kg was slightly lower than required by the standard [13]. The rest of the tested parameters met the requirements of the mentioned standard.

Using a laboratory blender, samples of fuel oil compositions containing significant amounts of HPO (30 and 50 wt %) and 70 and 50 wt % of EP were prepared. Due to the content of light fractions lowering the flash point, HPO with distilled out gasoline fraction (i.e., FHPO, the properties of which are presented in Table 2) was also used for fuel composition. The basic functional parameters of light heating oil compositions are presented in Table 3.

Because HPO contains light hydrocarbon fractions (as confirmed by the analysis of the composition of 30% of HPO with 70 wt % EP), the addition of hydrorefinate lowered the flash point below the requirements of the Polish standard (56 °C) [13]. This makes it impossible to use it for composing light heating oil. Therefore, fuel compositions with hydrorefinate devoid of gasoline fraction (FHPO) were prepared. The composition containing 30 wt % of FHPO met the tested requirements of the standard [13]. The sulphur content approached the accepted limit. On the other hand, the composition containing 50 wt % of FHPO did not meet the sulphur content (the measured value exceeded the threshold of 0.1 wt %) or specific weight requirements (it slightly exceeded the permitted level of 860 kg/m^3^).

The elemental composition of CPO, HPO and for comparison EP was determined. The results of the analysis are presented in Table 4.

The content of certain elements in CPO and HPO varied. First of all, the content of total sulphur in HPO was almost eight times lower than in CPO. This was the effect of hydrorefining. The chlorine content was also significantly reduced, and it dropped by a factor of three. In HPO the content of metals was also lower—in some cases by half. The content of elements to be determined in HPO was higher than in commercial EP oil. However, these were not drastic differences. The addition of HPO to the composed fuel oil would not significantly increase the emission of solid particles with exhaust fumes.

## 4. Conclusions

Due to its high total sulphur content and other unfavourable properties, pyrolytic oil obtained as a result of tyre pyrolysis is not suitable for use as motor or heating fuel. Therefore, pyrolytic oil with total sulphur content of 1.102 wt % was hydrorefined in the presence of a nickel–molybdenum catalyst deposited on alumina carrier (NiMo–Al_2_O_3_) at 380 °C and hydrogen pressure of 3 MPa. As a result of hydrogenation, hydrorefined oil with sulphur content of 0.145 wt %, double-reduced content of unsaturated hydrocarbons and significantly reduced content of aromatic compounds was obtained.

Due to its properties, hydrorefined oil was used as a significant component of the composition of light heating oil, which may meet the requirements of the Polish standard for L1 light oil [13]. For this purpose, 30 wt % of hydrorefinate was mixed with 70 wt % of Ekoterm Plus commercial light heating oil, with a total sulphur content of 0.072 wt %. Certain product properties were investigated. It turned out that the sulphur content and other tested parameters complied with the Polish standard, with the exception of the too-low flash point in a closed cup (<56 °C).

Therefore, the gasoline fraction (boiling point up to 180 °C) was distilled out from the hydrorefinate. The residue, with flash point 74 °C and sulphur content of 0.143 wt %, was used to obtain light heating oil. Two compositions containing 30 and 50 wt % of heavier fraction of hydrorefinate and the rest composed of Ekoterm Plus were prepared.

It was found that the fuel containing 30 wt % of the heavier fraction of hydrorefinate met the requirements of the standard for the tested parameters. The total sulphur content was 0.092 wt %, specific weight was 856 kg/m^3^ and the flash point in a closed cup was 64 °C. Parameters, which usually do not comply with the requirements of the standard, were met in this composition.

On the other hand, the composition containing 50 wt % hydrorefinate did not meet the requirements for sulphur content—the measured value exceeded the threshold of 0.1 wt %, and specific weight slightly exceeded the permitted level of 860 kg/m^3^.

Sulphur content, specific weight and flash point are the main parameters limiting the possibility of using hydrorefined oil from tyre pyrolysis for composing light heating oils compliant with the Polish PN-C-96024:2011 standard.

## Figures and Tables

**Table 1:** Hydrocarbon compositions of HPO and CPO.

Hydrocarbons	HPO	CPO
Alkanes, wt %	27.74	11.86
Dienes, wt %	3.42	7.67
Cycloalkanes, wt %	9.12	4.47
Cycloalkenes, wt %	2.11	3.73
Cycloaliphatic-aromatic hydrocarbons, wt %	5.14	5.64
Total aromatic hydrocarbons, including, wt %:	34.88	47.65
-benzene, wt %	1.61	2.23
-toluene, wt %	3.03	3.92
-xylenes, wt %	3.80	4.76
-ethylbenzene, wt %	2.61	3.31
-cumene, wt %	0.57	0.76
-styrene, wt %	0.22	0.95
Oxygen-containing compounds, wt %	0.16	0.82
Macromolecular compounds, wt %	0.93	1.84
Unidentified compounds, wt %	16.5	16.3

**Table 2:** Basic physicochemical properties of CPO, HPO, fraction of hydrorefinate (FHPO), Ekoterm Plus (EP) and requirements according to Polish standard [13].

Parameters	CPO	EP	HPO	FHPO	Requirements for Light Heating Oil L1 [13]	
					Min.	Max.
Density at 15 °C, kg/m^3^	921	837	874	894	-	860
Kinematic viscosity at 20 °C, mm^2^/s	5.82	3.61	3.42	5.76	-	6.0
Kinematic viscosity at 40 °C, mm^2^/s			2.27	4.03	-	-
Flash point (closed cup), °C	52	64	35	74	56	-
Flash point (open cup), °C	64	79	53	89	-	-
Solidification temperature, °C	−48	<−43	−36	−26	-	-
Fractional composition						
initial boiling point, °C	63		74	171		
distils to 250 °C, vol %	45	50	42	36		65
distils to 350 °C, vol %	84	94	88	93	85	
final boiling point, °C	353		356	348		
Sulphur content, wt %	1.102	0.072	0.145	0.143	-	0.10
Heat of combustion, MJ/kg	41.3	40.3	42.6	42.8	42.6	-

**Table 3:** Basic physicochemical properties of the compositions studied.

Parameters	30 wt % HPO 70 wt % EP	30 wt % FHPO 70 wt % EP	50 wt % FHPO 50 wt % EP
Density at 15 °C, kg/m^3^	848	856	866
Kinematic viscosity at 20 °C, mm^2^/s	3.47	3.92	4.69
Kinematic viscosity at 40 °C, mm^2^/s	2.32	3.43	2.02
Flash point (closed cup), °C	36	64	65
Flash point (open cup), °C	57	81	83
Solidification temperature, °C	−41	−38	−35
Fractional composition			
initial boiling point, °C	75	96	87
distils to 250 °C, vol %	51	43	46
distils to 350 °C, vol %	92	94	93
final boiling point, °C	353	353	351
Sulphur content, wt %	0.095	0.092	0.117
Heat of combustion, MJ/kg	43.85	43.20	41.55

**Table 4:** Elemental composition of CPO, HPO and EP.

Element	CPO (wt %)	EP (wt %)	HPO (wt %)
Al	0.0007	0.0004	0.0005
Si	0.0025	0.0012	0.0017
S	1.1020	0.0720	0.1450
Cl	0.0026	0.0005	0.0008
Ti	0.0002	0.0002	0.0002
V	<dt	<dt	<dt
Cr	<dt	<dt	<dt
Fe	0.0039	0.0008	0.0023
Zn	0.0008	0.0002	0.0005
Sn	0.0019	0.0003	0.0012

dt—detection threshold (about 10 ppm).
